# Estimating the allocation of land to business

**DOI:** 10.1371/journal.pone.0288647

**Published:** 2023-08-02

**Authors:** Michiel N. Daams

**Affiliations:** 1 Department of Economic Geography, Faculty of Spatial Sciences, University of Groningen, Groningen, the Netherlands; 2 Rudolf Agricola School for Sustainable Development, University of Groningen, Groningen, the Netherlands; ICIMOD: International Centre for Integrated Mountain Development, NEPAL

## Abstract

This paper is uniquely focused on mapping business land in satellite imagery, with the aim to introduce a standardized approach to estimating how much land in an observed area is allocated to business. Business land and control categories of land are defined and operationalized in a straightforward setting of pixel-based classification. The resultant map as well as information from a sample-based quantification of the map’s accuracy are used jointly to estimate business land’s total area more precisely. In particular, areas where so-called errors of omission are possibly concentrated are accounted for by post-stratifying the map in an extension of recent advances in remote sensing. In specific, a post-stratum is designed to enclose areas where business activity is co-located. This then enhances the area estimation in a spatially explicit way that is informed by urban and regional economic thought and observation. In demonstrating the methodology, a map for the San Francisco Bay Area metropolitan area is obtained at a producer’s accuracy of 0.89 (F1-score = 0.84) or 0.82 to 0.94 when sub-selecting reference sample pixels by confidence in class assignment. Overall, the methodological approach is able to infer the allocation of land to business (in km^2^ ± 95% C.I.) on a timely and accurate basis. This inter-disciplinary study may offer some fundamental ground for a potentially more refined assessment and understanding of the spatial distribution of production factors as well as the related structure and implications of land use.

## Introduction

Land is a non-negligible input to business. In advanced economies, land and intertwined built-up structures that are work-related represent roughly 15 to 35 percent of the value of all produced assets on national balance sheets [1]. Explanations of why land in particular locations is put to a business use are strongly established in modern economic analysis. These established explanations are primarily concerned with competition for space and land pricing as well as with land use structures in terms of urban function and density, in particular at the firm-location level [2–5]. At the more aggregate level of cities or regions, however, there is no systematic knowledge of how the quantity of land that is put to a business use may feed back into the economics of how well places perform, and neither so in terms of environmental or well-being impacts. One reason for this is that relevant information which is consistent across places as well as available on a timely basis is commonly absent. At present, the scientific assessment of business land is mostly confined to inconsistent definitions of land use across local zoning plans or to map information that is large-scale but limitedly available [6, 7]. Importantly, area estimates for business land, whether captured in tabular statistics or maps, are widely unavailable or updated only at relatively slow multi-year intervals, even in data-rich countries such as the United States where land use monitoring institutions are strong [8, 9]. This highlights the relatively fundamental relevance of being able to better scientifically observe how business land–as a long-recognized and capital-intensive factor of production [1, 10]–is allocated across cities and regions.

In this context, the rationale for adopting remote sensing is straightforward and similar applications show in particular in the fields of urban and regional economics [11–13]. These fields study the allocation of economic activity across space as well as patterns in the use of land, and the potential of methodological integration with remote sensing was already remarked by Nobel laureate Paul Krugman several decades ago [14]. Even so, the actual inter-disciplinary integration of urban and regional economic analysis with remote sensing analysis is presently very much still in its infancy. Also from a policy perspective, considering for instance the viewpoints of various international organizations including the World Bank, the OECD, or the European Commission [9, 13, 15, 16], or UN’s Sustainable Development Goal no. 11, further mutual efforts from the involved disciplines appear to be warranted [13]. Such relevant cross-fertilization of disciplines, as the assessment of business land allocations requires, is what the present study newly provides.

This study’s novelty embeds in two broad and consequentially related observations. First, what is apparent from studies that apply the remote sensing of cities or regions in the economic domain is that the spatial distribution of economic factors tends to be inferred only indirectly. Rather than defining conceptually what type of land cover features provide a relevant economic signal, in order to then map these features, the involved studies instead lean towards black-box type of approaches that predict local economic circumstances from conceptually limitedly defined patterns in land cover [17–22]. Although these approaches are remarkably useful for highlighting otherwise hard to observe variation in local economic development across administrative areas [23], whether any of the observed land is used for business or for other purposes remains inherently implicit as this is not conceptually defined nor measured directly.

Second, although in the remote sensing literature, on the other hand, studies typically define explicitly what type of land cover features are sought to be mapped in the observed imagery [24–28], studies that dedicate their empirical precision specifically to examining business land’s areal coverage have remained absent. Approximately nine out of ten remote sensing studies which explicitly consider urban or built-up land do target only a single class of such land, meaning that only mostly general patterns in developed land are examined, as a recent literature review shows [29]. The few remote sensing studies that do adopt more disaggregate classifications of developed or built-up land tend to focus on residential or infrastructural uses, although some studies consider blends of commercial, industrial, or otherwise ‘non-residential’ land use complementary to other map classes of their interest [30, 31]. As such, any specialized remote sensing methodologies that target business land and can be applied on a timely basis are lacking.

These overarching observations combined further underline that the geography of the urban or regional allocation of land to business is largely left open to enquiry across many places. This then warrants the development of a standardized methodology for the timely estimation of *how much* land in an observed city or region is allocated to business, which is precisely what this study aims to do.

This study introduces a methodology to quantify the allocation of land to business in terms of square kilometers within an observed city or region. The first step to this end is to map land that appears to be in a business use. To do so, a parsimonious definition of business land and relevant other ‘control’ classes is developed, which allows for a straightforward separation of different types of land cover and land use. The observed classes of land are predicted in Sentinel-2 satellite imagery using a tree-based classification model. The classification model operates at the level of individual pixels, which ensures a useful degree of conceptual clarity in separating different map classes from each other [28]. Alternative ‘object-based’ approaches based on deep learning have proven advantages for different albeit related classification targets, such as local climate zones [32], functional zones [33–35], built-up areas [36], or building footprints [37] in terms of classification accuracy [36]. However, the assignment of pixels to such map classes is then based on spatial context which may complicate the interpretation or cross-study comparison of accuracy outcomes [28, 29, 38, 39]. Further discussion of remote sensing classifiers is provided in a recent literature review [29]. To maintain class-definitional straightforwardness while also limiting dependence on the availability of reference data for different places [9], this study relies on relatively conventional classification and estimation practices that are well-established [40, 41] and applies these in a cross-sectional setting. The final estimation of business land’s total area is achieved using a sample-based procedure that accounts for mapping errors, which are inherent to any map classification. This then provides an area estimate that is more refined than when inferred directly from the map [41].

Relevantly, the present study’s discipline-blending novelty, beyond its thematic integration of fields, in a more detailed empirical sense draws on an economically informed extension of recent advances in remote sensing-based area estimation for rare map classes. These recent advances entail a post-classification modification of the observed map as to include an additional stratum, which then reduces the possible influence that errors of omission may have on the accuracy of area estimates [42]. The involved prior studies have considered omissions for a map category of deforestation [43–45]. Their post-strata seek to efficiently enclose map areas that include concentrations of pixels that are incorrectly mapped as forest instead of as deforestation. In specific, in those studies map pixels are assigned to the post-stratum based on buffers around mapped instances of deforestation, their class of interest. To generate such buffers various buffer-lengths consisting of one to several pixels have been used in the different studies and the hypothetical effects this has on the accuracy of area estimates has been explored in [40]. In the case of business land, however, the spatial dimension of the post-stratum can be based on urban and regional economic domain knowledge. This specifically regards the well-understood stylized fact that businesses may cluster in space [5]. As such, in the present study the map is post-stratified along a measure of local business clusters. These clusters can be of any shape or size, and allow the map’s post-stratification to be straightforward as well as agnostic to study area dimensions. This additional conceptually informed empirical feature of the study may further ensure and enhance the precision at which the area of business land is estimated.

To illustrate the methodology for estimating how much land is allocated to business, the San Francisco-Oakland-Berkeley Bay Area is examined. This study area covers a 6,609 km^2^ surface, has 4.6 million inhabitants according to the 2021 US census, and encloses several cities along with the wider metropolitan region. The metropolitan region covers sub-areas of varying settlement structures that are within commuting range from the cities and so includes both central and decentral patterns of land use, as well as ‘dispersed’ concentrations of business activity in the form of strings of industrial clusters throughout Silicon Valley. The diverse urban structure of the Bay Area, as described and documented richly in various other studies [46–48], altogether offers a meaningfully varied geographical ground for this paper’s purposes.

The remainder of this paper is organized as follows. The next section introduces the conceptual definition and empirical operationalization of business land and the observed control categories of land use and land cover. The subsequent section presents the results for the mapping and area estimation exercises, which will show that the allocation of land to business can be estimated with marked accuracy, and is followed by brief conclusions and further discussion.

## Methods and data

### Class definitions and sampling

The class of interest is land allocated to business. Business land is defined as land that is developed, whether flatly or covered by buildings or other vertical structures, and predominantly in use for business purposes. This follows how land is typically categorized in economic analyses of land use [4, 6, 7]. In effect, the definition includes sites covered by, for instance, offices, stores, small or corporate businesses, parking lots for workers or clients, warehouses, or structures used for storage of goods or industrial production. This largely resembles the definition of commercial and industrial land in the well-established Anderson Level II classification [24], although that classification pragmatically allows for a blending-in of residential land or other non-business land to some extent, whereas in the present study separation is stricter.

To separate business land from other types of land effectively and parsimoniously, also a select set of mutually exclusive ‘control’ classes that are essentially unrelated to business is observed. In specific, any land unrelated to business is categorized as either ‘developed otherwise’ or ‘non-developed’. The class of ‘other developed land’ pertains to land that is covered by built-up structures such as, for instance, houses, schools, recreational amenities, or paved infrastructure that includes roads as well as parking lots and sidewalks. ‘Non-developed land’ is land that is not covered by man-made materials or structures and so includes, for instance, forests, agricultural fields, vegetated gardens, and water bodies. This typology ensures that the class of land which resembles business land most closely only has a limited areal weight in the study area, which is useful to area estimation as will be discussed further shortly. Now that classes are defined, we can turn to their operationalization in terms of sampling.

The sample for model training consists of 2,489 pixels that are classified visually based on Google Earth imagery. Google Earth imagery’s resolution provides a reference for the period of study that is more granular than the spatial unit (10 x 10-meter pixels) of the observed imagery. This allows the visual classification of samples to be based on reference information that is of a superior quality [41]. Although Congalton [49, p.35] notes in a seminal paper that “*collecting reference data may be as simple as obtaining a county zoning map*”, zoning maps are of little use here as these typically rely on local definitions that vary across sub-regions whereas for this study’s purposes consistent class definitions are more desirable. During visual assessments of the sampled pixels, the within-pixel proportion of the observed class is considered. When multiple classes are present, class-assignment adopts the class that covers at least half of the pixel, following [50] and [51]. In the case of a pixel in which a quarter to half of the pixel encloses a class other than the assigned class, the class assignment is recorded as one for which the confidence in class identification is limited. These pixels remain in the sample to account for, rather than ignore, any complexity in the observed configurations of land [52]. The measure of confidence captures that identification of the correct class is potentially confounded by a mixed nature of the area which a pixel encloses, or by some residual doubt about the correct class. Class assignment, however, is straightforward as the parsimonious definition of classes allows for any classes to be disentangled effectively. To further ensure consistency of classification and to account for possible clerical errors, each of the individual samples is revisited. In instances for which further site assessment is possibly useful, this is resolved decisively by using Google Street View, which has comprehensive coverage across the study area’s urban surface. This is an advantage over many other remote sensing studies which may occasionally warrant on-site inspection of some of their samples to ensure correct class assignment in relatively remote locations. Prior to assigning the samples to classes it has to be considered, however, that the class of interest is somewhat rare.

Whereas cities are typically composed mostly of land that is in a residential use, in terms of area proportion business land is less abundant. Therefore, to ensure that business land is appropriately sampled rather than under-sampled, see [49] or [41] for a general discussion of this issue, stratified random sampling is used. The required stratification of the study area is obtained from a combination of developed areas according to the U.S. Geological Survey’s 2019 National Land Cover Database with information about the distribution of jobs at the granular level of census blocks (see S1 Fig). The jobs data are sourced from the U.S. Bureau of Labor Statistics and draw on mandatory worker insurance records. These records cover the universe of primary work locations in our study area. This type of information, other than information on local zoning boundaries, has the advantage of being available on a consistent basis across different locales within the study area. The resultant strata designate the study area as 283 km^2^ (4.3%) of land that is likely business-related, 1,251 km^2^ (19.0%) as otherwise developed land, and 5,069 km^2^ (76.7%) as non-developed land, all in a priori terms. This then serves as a basis for the allocation of sample units.

Sample units are allocated using a so-called ‘compromise’ approach [41]. This approach addresses that business land’s limited area proportion would otherwise lead to only few samples being drawn in an area-proportional sampling design, by shifting the number of samples towards an allocation that is equal between the observed classes. This intends to balance the user’s accuracy and producer’s accuracy of the final classification of business land. User’s accuracy reflects errors of commission by incorrectly assigning pixels to the observed class, whereas producer’s accuracy reflects errors of omission, meaning that pixels that should have been assigned to the observed class were assigned to another class. These accuracies in part respond to sample size.

In determining sample size, a reasonable starting point from which to grow the total sample is obtained in line with recommendations in the literature [41]. More precisely, suggested sample sizes are obtained from a standard equation for stratified random sampling, Eq (5.25) in [53]. This equation is estimated in 10,000 iterations that each consider a different set of hypothetical input values with regard to classification accuracy targets. The resultant distribution of suggested sample sizes is described by mean, standard deviation, and maximum values of approximately 130, 300, and 2,300, respectively. Only about one in ten of the suggested sample sizes is in excess of 250 samples in total, which is used as a reasonable starting point. Further samples for each class are added incrementally based on improvements in model performance, similar to [40] and as shown in S2 Fig, until classification accuracy is stable. This then obtains the final sample for training the classification model.

### Classification model and imagery

To predict the class for each pixel in the observed satellite imagery, an established tree-based model is used, the random forest model [54]. The model grows an ensemble of individual decision trees that each partition the training sample into classes down a hierarchy of branches, with an observed pixel’s final class assignment being based on majority vote across the ensemble. The estimation relies on a limited number of parameters and is robust to overfitting [55]. The number of trees is set to 1,000 as generally desirable [54] and no further assumptions are imposed on the model’s form [56]. Parsimony is preferred, considering also the model’s general effectiveness at classifying urban areas in satellite imagery [50].

The observed imagery come from the Sentinel constellation of satellites. These satellites, operated by the European Space Agency under the European Commission’s Copernicus program for Earth observation, offer imagery at pixel resolutions as granular as 10 x 10 meters. This allows for relevant local urban features to be captured. In specific, bottom-of-atmosphere Sentinel-2 imagery (Level-2A) is observed. The full set of the atmospherically corrected imagery’s spectral bands is complemented with Sentinel-1 synthetic aperture radar imagery, specifically the VV and VH bands as these may capture further aspects of urban structure [31, 57], as well as with slope information derived from a same-resolution USGS digital elevation model as built-up areas other than some non-built-up areas tend to be relatively flat [58]. These data, in pre-processed form and in a consistent projection, are obtained using Google’s Earth Engine platform [59].

In total, 84 million pixels are observed, where each pixel captures pixel-level median values for image bands as obtained over all images available over the period of study, which is 2019 consistent with the reference data’s dating, after masking away clouds and cirrus in Sentinel-2 imagery using its QA60 band to prevent obfuscation of land features by clouds or shadows. The resultant cloud-free imagery is assessed visually [41] and no obvious errors were found. Finally, two common indices that capture normalized differences in land cover by vegetation or built-up areas are included, namely NDVI and NDBI, respectively. Discussion of these indices is provided in [50]. Additionally, whereas some studies use texture metrics [60, 61], possible dispersion in NDVI values for all directly neighboring pixels is captured in one additional band. This may possibly help to capture some locational structure which sets business features apart from non-business urban features that are more fine-grained in terms of land cover mix, such as sub-urban homes surrounded by gardens. These additional indices may offer some further reassurance that the classification model is informed about differences in land cover that are relevant to generating the initial map classification.

### Post-stratification of the map

The map’s informativeness for sample-based estimation of business land’s total area is enhanced by extending the business land stratum through post-stratification. Post-stratification here accounts for the area of land allocated to business typically being relatively limited compared to the areas of undeveloped land or land that is put to a use other than business. Otherwise, estimates of the area of business land may be underestimated or, more particularly so, exacerbated, as other studies suggest in the context of forest cover which in area proportion terms is also a rare class [42–45].

The underlying issue is that omissions for a rare class, as observed in a sample of pixels used to estimate the class’s area, could be concentrated in particular sub-zones within the map area of a relatively abundant class. These errors would in area estimation be associated with their proportion in the total area of the abundant class from which the omissions are retrieved, whereas the smaller area of the sub-zones in which the errors are concentrated would be more relevant to use. This could in part be addressed by drawing a larger sample and stratifying it along the original strata but, even then, uncertainty regarding the efficiency of that sample’s stratification would remain. An efficient approach to address this, as [42] discuss in detail, is to post-stratify the map by designating separate map strata for sub-zones where concentrations of omission errors are plausible. Doing so orients the sampling of pixels for map validation and area estimation towards sub-zones in the map where omission errors are likely, while the involved subdivision of larger strata into smaller (post-)strata lowers the weight of omission errors in the area estimates for the rare target class. This then improves the certainty around area estimates, especially when the empirical design trades off the ability to capture omission errors against a post-stratum’s map area proportions efficiently. As such, post-stratification should closely delineate those areas in the map where errors of omission for the class of interest are concentrated.

**Fig 1:**
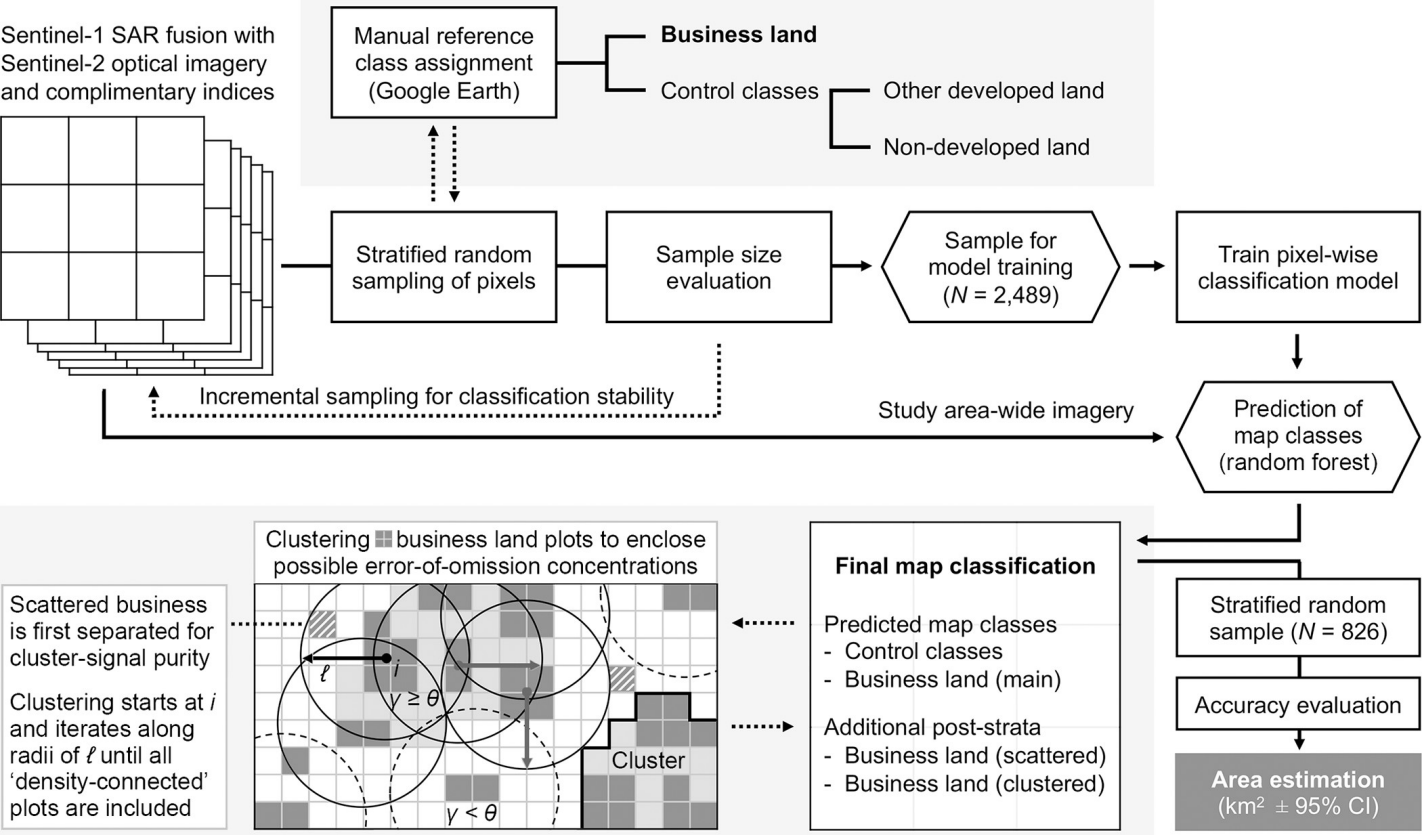
Schematic visualization of the methodological approach.

In the case of business land, foundational domain knowledge suggests that to efficiently spatially enclose errors of omission in post-strata, it can be considered that businesses tend to concentrate in local clusters [5, 62–64]. This stylized fact originates largely from market forces, such as transport costs and knowledge spillovers, although the clustering of business activities may be further enforced by zoning policies. For these reasons, a comprehensive post-stratum of local business clusters is generated to enclose land that is either used for business or located close to land that is put to use for business. As such, areas where concentrations of omission errors are plausible are accounted for considering the various forces that make business activities co-locate. This empirical step’s positioning within the overall methodology is illustrated in Fig 1.

Before generating any clusters, however, pixels that are mapped as business land but which may reflect relatively fragmented small-scale land cover features are eroded away a separate post-stratum of ‘scattered’ business land (Fig 1). In specific, this re-assigns any pixels that are not part of a block of at least 5 *x* 5 adjacent business land pixels (50 *x* 50 meters), which serves as a bottom threshold in mapping the dimensions of plots of commercial land and related built-up structures. Smaller-scale mappings reflect that pixels are conceptually more likely to belong to a map class other than business land (cf. class definitions) as will be confirmed also empirically from class assignment probabilities shortly. The map refinement mostly addresses patterns of land use in medium-density urban areas. There, other than in mostly business-related areas, built-up structures are relatively scattered across space, as tends to be observable in areas in between central business districts and suburbs. Accounting for this allows clusters to be generated from a relatively pure main stratum of business land, which makes the clusters even more area-efficient as relevant to sample-based area estimation [cf. 42].

Clusters of business land are obtained using a well-established algorithm, namely Density-Based Spatial Clustering of Applications with Noise (DBSCAN), which is remarkably useful for the empirical definition of clusters [65, 66]. This algorithm is agnostic to city-size and clusters can be of any shape or size. In fact, the approach considers that clusters of business activity are essentially clusters-within-clusters, as any city in which the cluster is located is in essence a cluster itself as well [67]. As such, in obtaining intra-city clusters the algorithm observes only plots of business land that are surrounded within observed radius ℓ by at least the expected amount *λ* of business land as when spread evenly across the study area in the hypothetical situation of any clustering’s absence [68].

Under this condition a first cluster is initiated, as illustrated in Fig 1, from the centroid *i* of a randomly selected plot of business land as observed after merging any adjacent pixels assigned to this category. As Fig 1 illustrates as well, per convention for two-dimensional data, the number of business land plot centroids surrounding centroid *i* within radius ℓ should meet a threshold cluster size *γ* of *γ*≥*θ*, where *θ* = 4 [65]. These nearby plots are then grouped together and from that ‘core’ group the cluster is grown, iteratively, in an outward direction by adding-in *any* further plots whose centroids are ‘density-connected’ to the plots already in the cluster, for being within ℓ meters away, until no further such additions to the cluster can be made. Further clusters are grown reiteratively until all clusters are obtained. This process does not require that all observed plots of business land are assigned to a cluster, nor is there any overlap between clusters, and there is no a priori estimation of the appropriate number of clusters. Concave hulls delineate the spatial shape of each individual cluster (Fig 1). Distinct sets of clusters are generated separately using ℓ values between 0 and 1,000 meters in 50-meter increments. Selection of the final set is based on maximizing both the inclusion of business land plots and the clusters’ total area, subject to *λ*. The latter condition ensures that the clustering process does not naturally converge towards the entire city being identified as a cluster. The resultant cluster-based stratum as integrated into the final map includes only within-cluster pixels that priorly were associated with either scattered business or map classes *other* than business land. Therefore, pixels in business land’s main stratum, when located within clusters, are surrounded but not absorbed by the clusters. As such, the clusters represent ‘tissue’ between plots of business land where, according to theory and stylized facts [cf. 5, 62–64], omission errors are likely to be concentrated in the map.

### Map validation and area estimation

For map validation a stratified random sample of 826 pixels is drawn from the inferred map. This additional sample is categorized using the same approach as for the training sample. Sample allocations to each *i*th map class, as well class-specific areal map weights *W*_*i*_, can be observed from Table 1. Using this sample, the agreement between the map and the reference data can be assessed from cross-tabulation in an error matrix. Based on this information, map accuracy can be quantified in overall terms as well as, more importantly, in terms of user’s accuracy and producer’s accuracy, which are standard metrics that reflect the map classification’s errors of commission and errors of omission, respectively, for each of the observed map classes separately [41]. This allows for evaluation of the empirical strategy’s performance at mapping business land in specific.

**Table 1:** Description of the (post-)strata as well as the stratified random sample of pixels used in map validation and for sample-based area estimation.

	Business Land			Control Categories	
	Main	Clustered	Scattered	Other Developed	Non-Developed
*W*_*i*_	0.019	0.012	0.011	0.143	0.815
*n*_*i*_	90	87	87	142	420

*Notes*: *n*_*i*_ denotes the allocation of a sub-sample to the *i*th observed category of land use or land cover, for the sample (*N* = 826) that is used in map validation. A compromise allocation is used, as obtained from averaging area-proportional and equal allocations. *W*_*i*_ gives the map area weights based on pixel counts. Business land strata are obtained based on post-stratification. In summary, the post-stratum of scattered business land includes a sub-set of pixels that were mapped as business land but which are held separate for being relatively scattered across space. This enhances the spatial signal of the main business stratum as the post-stratum of local business clusters is generated. The clusters, by design, enclose possible concentrations of omission errors, which provides an efficient basis for the sample-based estimation of the total area of business land within the study area.

Using the error matrix also the total area of business land within the study area is estimated. This estimation considers the proportions of business land across all of the observed map strata, as readily adjusted for any validated classification errors. Therefore, these area proportions when multiplied with their stratum’s total map area, as observed from counting pixels while adjusting for possible areal distortion by the map’s projection, in sum provide a more appropriate area estimate than the map itself [40–44].

The area of map class *k* is estimated, following [41], as:

$${\hat{A}}_{k}=A({\hat{p}}_{∙k}),$$ (1)

where ${\hat{A}}_{k}$ denotes the *k*th class-specific area estimate; *A* represents the map’s total area; and ${\hat{p}}_{∙k}$ is the sample-based estimate of the observed class’s ‘true’ area proportion, which is computed as:

$${\hat{p}}_{∙k}=\sum_{i=1}^{5}W_{i}\frac{n_{ik}}{n_{i}.},$$ (2)

where *n*_*ik*_ is the number of pixels, amongst those that are sampled from the *i*th map stratum, that is correctly assigned to the *k*th reference class; *n*_*i*_. the row total which reflects the total number of pixels in the validation sample that is mapped to the *i*th map class; and *W*_*i*_ gives the area for map class *i* as counted directly from the map. In effect, this obtains the estimate for the map proportion of correctly mapped pixels for observed reference class *k* in each *i*th map stratum, including the post-strata, and then sums these area proportions. As such, area proportion ${\hat{p}}_{∙k}$ reflects the total area that is correctly mapped to reference class *k* across the map strata, which can then be multiplied with total map area *A* to obtain the observed class’s area estimate ${\hat{A}}_{k}$ (Eq 1). For each area estimate, a 95% confidence interval is approximated as:

$${\hat{A}}_{k}\pm 1.96[S({\hat{A}}_{k})],$$ (3)

where $S({\hat{A}}_{k})$ can be written as:

$$S({\hat{A}}_{k})=A\sqrt{\sum_{i}\frac{W_{i}{\hat{p}}_{ik}-{\hat{p}}_{ik}^{2}}{n_{i}.-1}},$$ (4)

where ${\hat{p}}_{ik}$ denotes the estimated map proportion $W_{i}\frac{n_{ik}}{n_{i}.}$ as given by error matrix cell (*i*, *k*). Eqs (1) and (3) are the equations of interest, as these give the sample-based area estimates as well as their degree of certainty.

## Results

**Fig 2:**
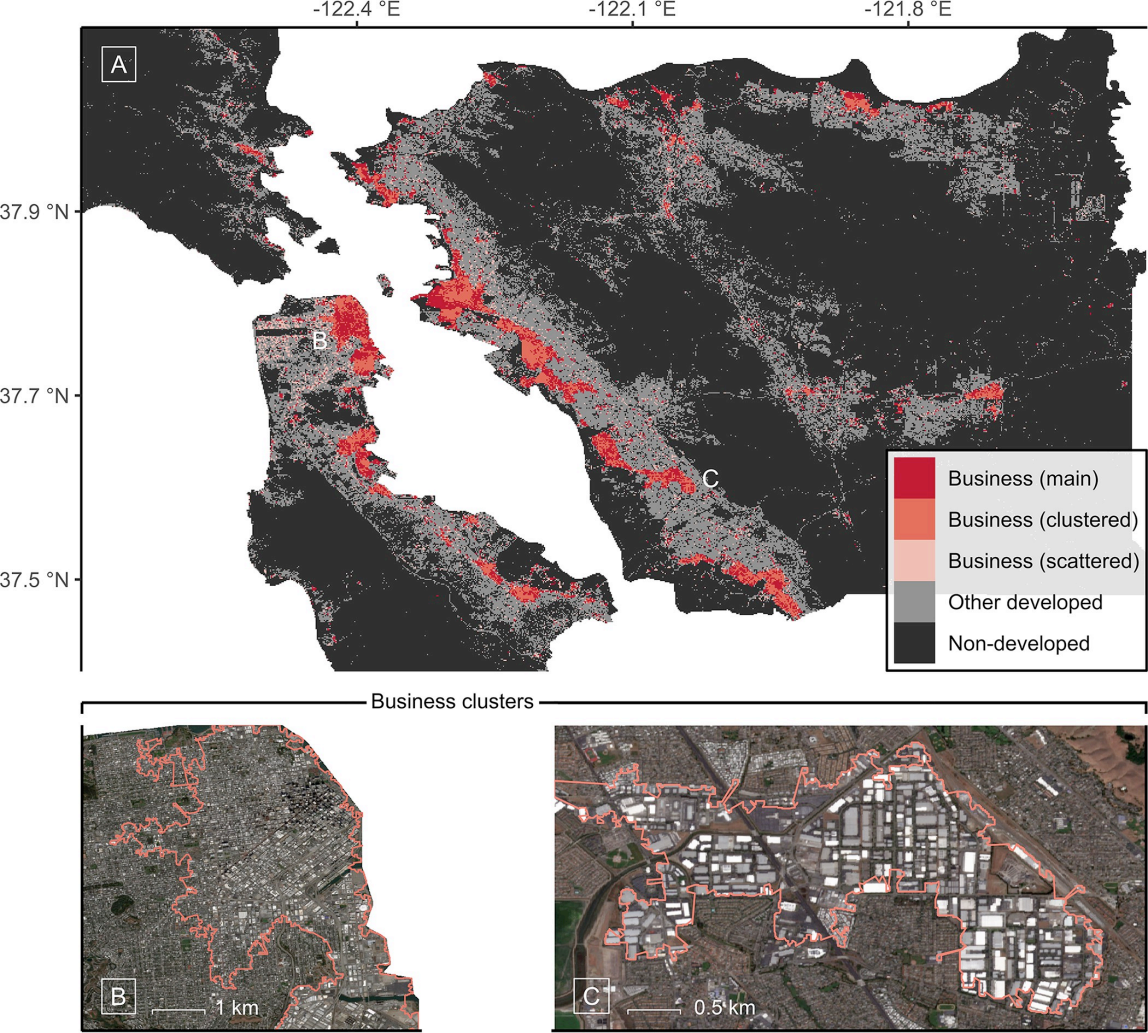
Classification of business land and associated local business clusters in satellite imagery covering the San Francisco-Oakland-Berkeley metropolitan area. (A) Classification of business land and control categories of land. (B) Boundaries of a relatively central business cluster in San Francisco and (C) a relatively sub-urban business cluster in Oakland, as both plotted on an RGB sub-set of Sentinel-2 imagery. Panels B and C illustrate how the post-stratum of clusters may capture spatial bundles of business land of any shape or size. Sentinel imagery are publicly available under the EU Copernicus Earth Observation Program.

The geography of business land as mapped across the study area can be observed from Panel A in Fig 2. As the map shows, business land is a somewhat rare class in terms of areal coverage. Across the San Francisco-Oakland-Berkeley metropolitan study area, business land is spread in a way that is both fragmented and characterized by dispersed concentration. Concentrations of business land are mapped from central San Francisco down to places such as Menlo Park, from Fremont up to Richmond, and across places towards the east such as Livermore. In line with the urban economic domain knowledge suggests [48, 62], a vast amount of business land, namely approximately two-thirds of all pixels in business land’s main stratum, is located inside clusters (*N* = 37).

The outer boundaries of the clusters obtained in post-stratifying the map are illustrated in Fig 2 Panels B and C. Apparent is that both the shape and size of the measure of clusters adapt well to different spatial settings. The cluster in Panel B broadly captures San Francisco’s central business district whereas the cluster in Panel C effectively encloses a center of business activity in a more decentral or sub-urban setting within Oakland. Across all individual clusters, mean areal size is 4.5 km^2^ with a standard deviation of 7.3 km^2^ and the combined area of the clusters is 165.4 km^2^. These cluster areas may in part include land that is in a use other than business, as business may be the predominant but not the exclusive use in these sub-areas within the map.

**Fig 3:**
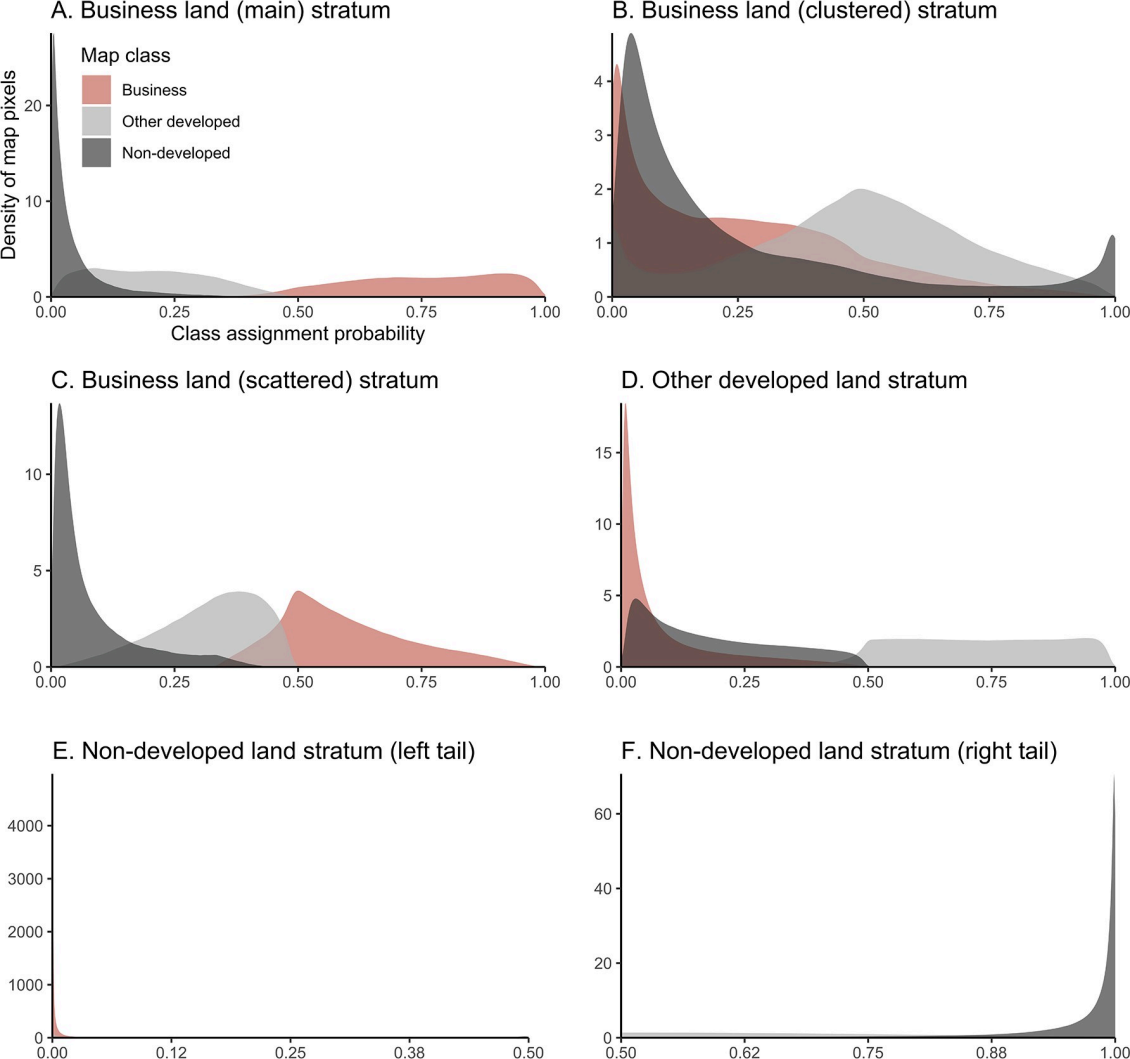
Distribution of pixel-level class assignment probabilities by map stratum. A. Business land (main) stratum, B. Business land (clustered) stratum, C. Business land (scattered) stratum, D. Other developed land stratum, E. Non-developed land stratum (left tail), and F. Non-developed land stratum (right tail).

Initial insight into the extent to which different map classes cohere or are separated from each other by the map classification model is provided in Fig 3. Fig 3 shows the distribution of posterior probabilities of class assignment, by stratum in the post-stratified map. The probability values range from 0 to 1 and are obtained at the pixel-level prior to the map’s post-stratification along scatteredness and clusters. Fig 3, Panels A to F, suggests that business land is well-separated from the control categories in the map, and is particularly separable from non-developed land. In contrast, land that is in any use other than business is much more intertwined with non-developed land. Fig 3 Panels B and C furthermore show that in their strata the distribution of probabilities of assignment to business land overlaps with the probability distributions for the other classes, which is in line with expectations. This relevantly reflects in the clusters stratum’s case that map pixels which are not mapped as business land are intentionally enclosed, as these may potentially harbor any map omissions for that category. Such can be further examined from the map classification’s accuracy in terms of its agreement with a manual reference classification.

The level of agreement between the map classification and the reference classification for a stratified random sample of map pixels is reported in Table 2. Overall, the reference classification underlines that business land is well-separated from the map’s other classes. Relevant errors of commission, which reflect instances of pixels that are mapped as business land but which should have been assigned to a different map class, can be traced mostly to land that is developed but which is in a use other than business according to reference information. For instance, along the rows of the error matrix in Table 2 it is shown for the main stratum of business land that 1.5% of the total study area is correctly mapped to the business land category, whereas 0.3% and 0.1% of total area should instead have been mapped to the categories of developed land in any use other than business or non-developed land, respectively. Such confusion of map classes is particularly descriptive of the stratum of scattered business land. Although the clusters-based stratum has a relatively mixed ‘true’ composition as well, this stratum includes other developed land and non-developed land by design.

**Table 2:** Classification accuracy and sample-based area estimates.

	Reference Categories		
	Business Land	Other Developed	Non-Developed
**Error matrix, map area shares**			
Business (main)	1.5%	0.3%	0.1%
Business (clustered)	0.4%	0.4%	0.4%
Business (scattered)	0.4%	0.6%	0.1%
Other developed	0.3%	11.5%	2.5%
Non-developed	0.0%	1.6%	79.9%
**Error matrix, sample counts**			
Business (main)	71	16	3
Business (clustered)	28	30	29
Business (scattered)	32	49	6
Other developed	3	114	25
Non-developed	0	8	412
**Accuracy and area estimates**			
Area km^2^	173.8	950.7	5,479.2
Area km^2^ ±	27.1	95.0	92.6
User’s accuracy	0.79	0.80	0.98
Producer’s accuracy	0.89	0.80	0.96
F1-score	0.84	0.80	0.97
Overall accuracy	0.93		

*Notes*: Map classes are listed by row. User’s accuracy for business land is obtained from this class’s main stratum. F1-scores give the harmonic mean of user’s accuracy and producer’s accuracy in the observed column. Area estimates and the associated 95% confidence intervals (km^2^ ±) are obtained using Eqs (1) and (3), respectively.

Map errors of omission on the other hand, which are instances of pixels that predominantly cover business land but are not retrieved as such from the map classification, are solely observed within the map stratum of other developed land. This is shown in the first column of the error matrices in Table 2. No such omissions are observed within the stratum of non-developed land, which is reasonable given the clear conceptual separation of this class and business land as underlined also empirically in Fig 3. Furthermore, the share of land that according to the reference classification should be assigned to business is expanded by approximately one-fifth of its original area when the tendency of businesses to cluster locally is accounted for through the map’s post-stratification. Partly as a result of this, the producer’s accuracy for business land is 0.89, which is higher than for other developed land and even rather close to the producer’s accuracy for the relatively large and more straightforwardly mappable class of non-developed land. This means that relatively many of the true instances of business land are actually retrieved from the observed satellite imagery, providing further reassurance that the sample-based estimation of business land’s area is well-informed.

Producer’s accuracy for business land is also examined for sub-sets of the reference sample for which class assignment is either clear or potentially confounded by a mixed nature of the area which a pixel encloses or some residual doubt due to other factors, taking values of 0.94 and 0.82, respectively. This underlines the relevance of including mixed pixels for which classification accuracy is limitedly certain in the analysis. Otherwise, producer’s accuracy and the underlying information which is relevant to area estimation may be (slightly) overestimated. For comparison, for other developed land, the producer’s accuracy for high-confidence and limited-confidence pixels is 0.87 and 0.25, respectively. Taken together, these findings suggest that the accuracy at which business land is separated from other classes is relatively robust.

The estimate of interest indicates that, across the study area, 173.8 km^2^ ± 27.1 km^2^ (2.6% ± 0.4%) of land is allocated to business (Table 2). For comparison, the area of other developed land (950.7 km^2^ ± 95.0 km^2^) is more than five times larger, whereas the majority of the observed metropolitan area’s land (5,479.2 km^2^ ± 92.6 km^2^) is not covered by any built surfaces or structures. Furthermore, the area of likely business-related land as per the map that was used to sample pixels for model training purposes falls outside of the area estimate’s 95% confidence interval, which additionally underlines the area estimation’s relevance. Whether the confidence interval’s width is sufficient ultimately depends on a study’s specific analytical objectives and this is not explored further here given the present study’s introductory nature.

## Discussion

This study has introduced a methodology that is uniquely focused on assessing business land from satellite imagery. The specific aim was to estimate the area land that is allocated to business in an observed region, and to do so on a timely and standardized basis. A demonstration of the methodology for the San Francisco Bay Area metropolitan area in the United States showed that business land can be mapped using a pixel-based classification approach in a parsimonious way and with marked accuracy. To achieve this accuracy, the classification approach has exploited the signal from initially mapped business land to generate a map post-stratum of local clusters of business. This spatially explicit definition of local business clusters was set out to capture and account for omission errors, which are inherent to any map classification, across the map in an area-efficient way. The clustering-based approach to post-stratification was confirmed through sample-based map assessment to contribute to area estimation accuracy. This underlines the usefulness of the methodology’s integration of recent advances within the field of remote sensing with the domain knowledge of urban and regional economics.

This study’s analysis of satellite imagery represents an inter-disciplinary step that may invite a further ‘modernizing’ of the empirical scientific observation of commercial land use. The use of land by businesses was long central to the field of economics, and has since various classical works remained as a modern textbook factor of production [5, 10, 69]. Yet, as highlighted also in [70], many possible complexities and processes can potentially be explored regarding the structure and performance of cities and regions, and these may be co-shaped by business land allocations.

What lines of scientific inquiry in this specific context are potentially the most fruitful remains fundamentally open. However, the monetary value of business land as a business asset is massively represented on bank [71] as well as national balance sheets [1], suggesting that renewed assessment of land and its geographic distribution as a production factor is promising. The modern economic literature has largely separated the understanding of business processes from discussions of related land supply, focusing more on production factors such as labor [72] and entrepreneurship [73]. Yet, all these matters relate to the built-up spaces that host firms and lock many processes into location [74]. As such, the allocations of land to business that can be observed using remote sensing are, more so than the literature might suggest, closely intertwined with many of the processes that economists are concerned with.

Debates that could potentially be further informed using this study’s methodology include those of the spatial structure of firm clustering [48, 62], given the city-size agnostic measurement of business clusters in satellite imagery, as well as the actually observable sources of inequality in urban development [13] that underly the nightlight reflections that are usefully analyzed in many studies [18, 75]. Neither is there any systematic knowledge regarding how the quantity of business land and its distribution across cities and regions relates to debates [76, 77] about sectoral structure in terms of specialization and diversification. This leaves room for further integrative assessments of how business land allocations arise as well as feed back into the economic performance of different places, as well as of how this relates to external impacts on the environment [25, 78] or the well-being of residents [79], which all is of increasing importance given persistent global processes of urbanization [80, 81]. As such, this study may offer some fundamental ground for the potentially more refined assessment and understanding of the spatial distribution of production factors as well as the structure of land use and its various implications.

## Supporting information

S1 Fig Area-efficiency of the business stratum for training pixel sampling. (TIF)

S2 Fig Model performance by sub-sample size. (TIF)

S1 File (DOCX)
