# Supplementary figures and images for: Estimating the allocation of land to business
Source: PLoS One. 2023 Aug 2;18(8):e0288647. doi: 10.1371/journal.pone.0288647 (PMC10396024; doi:10.1371/journal.pone.0288647)

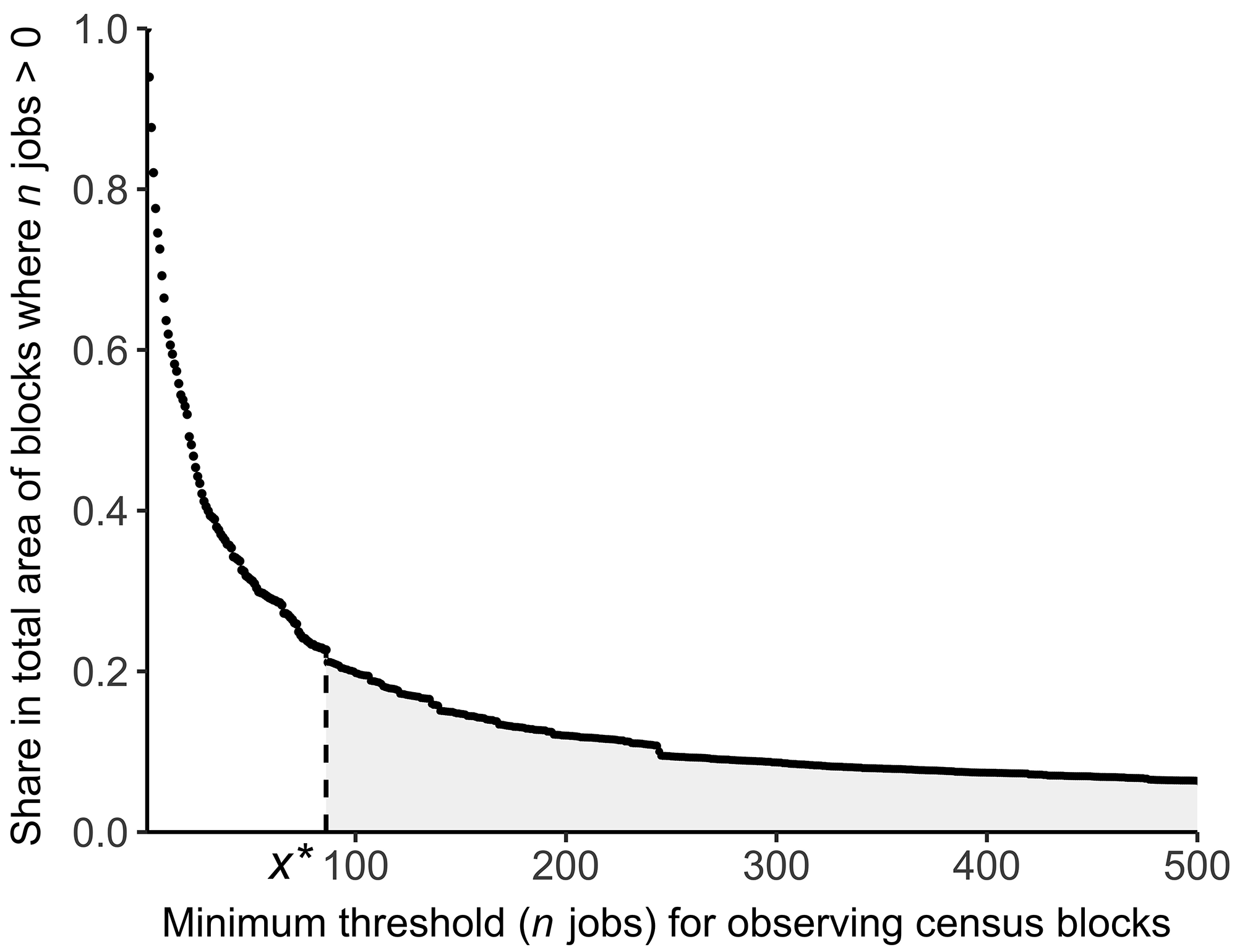

Supplement: S1 Fig — (TIF) [file pone.0288647.s001.tif]

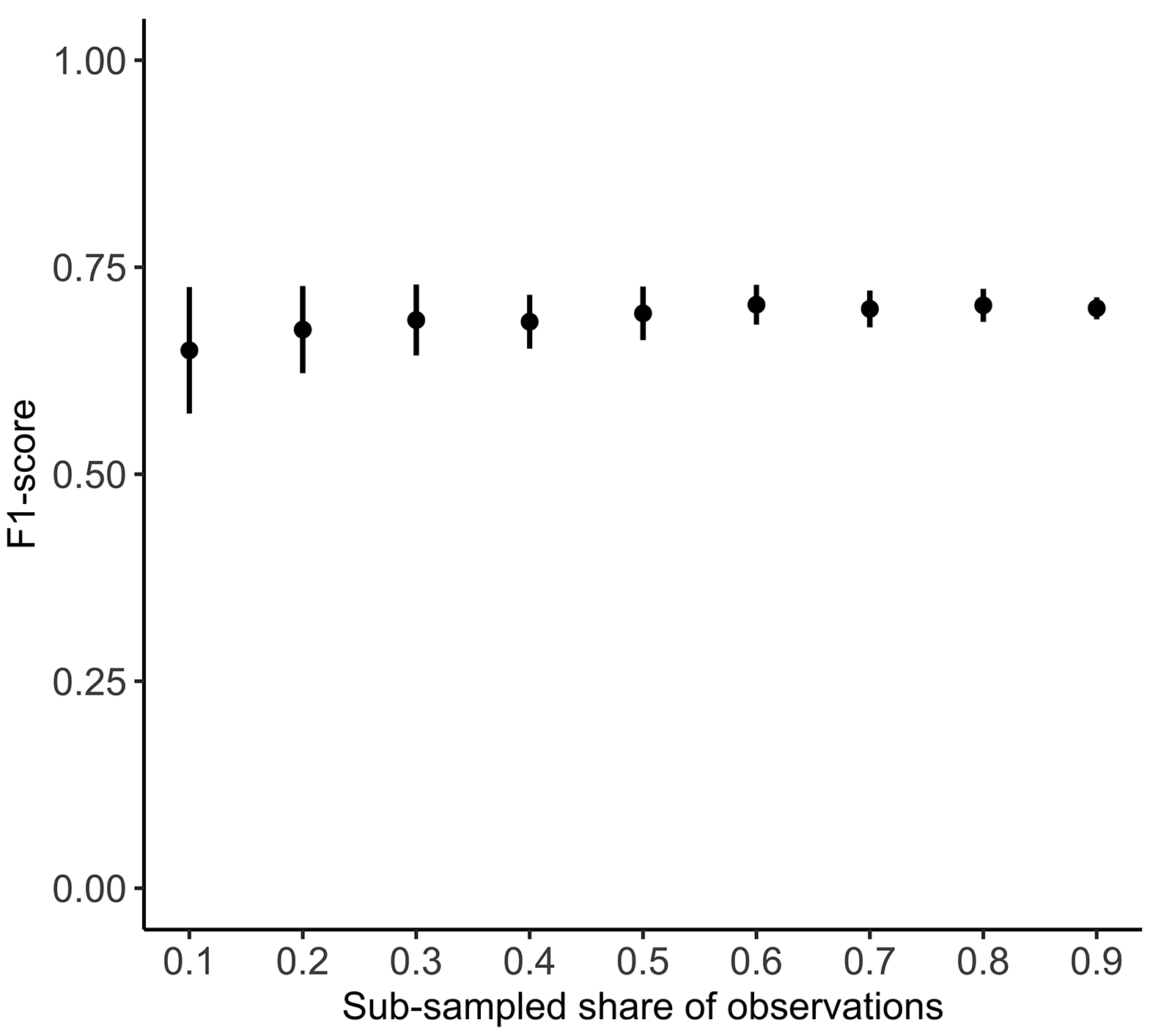

Supplement: S2 Fig — (TIF) [file pone.0288647.s002.tif]
