# Supplementary material for: Estimating the allocation of land to business
Source: PLoS One. 2023 Aug 2;18(8):e0288647. doi: 10.1371/journal.pone.0288647 (PMC10396024; doi:10.1371/journal.pone.0288647)
Supplement: S1 File — (DOCX) [file pone.0288647.s003.docx]

Supplementary information for the paper
“Mapping the Allocation of Land to Business”

Michiel N. Daams ^1, 2*^

^1^ Department of Economic Geography
University of Groningen, Groningen, the Netherlands

^2^ Rudolf Agricola School for Sustainable Development
University of Groningen, Groningen, the Netherlands

^*^ E-mail: m.n.daams@rug.nl

*I. A priori map stratification*

In urban areas, such as in this case the San Francisco-Oakland-Berkeley so-called core-based statistical area (6,609 km^2^), which encloses several cities and their wider metropolitan areas, employment is in part woven into sub-areas that are not particularly business-related but do cover vast amounts of land. To target instead those areas that are represented by land use features that signal business activity, such areas are delineated based on census blocks where the number of jobs is higher than a threshold value. Increasing the threshold value has ‘diminishing returns’ in terms of clearing away areas where the number of jobs is non-zero but also not of any distinctive amount. The threshold is determined, as shown in Figure S1, by the value after which the gains (a ‘purer’ business stratum) become limited relative to the costs (omission of areas that are less and less sparse in terms of business activity). This resembles common practice in the field of machine learning [S1] and, in the absence of precise theoretical guidance, this approach is relatively efficient and also considerate of the very nature of cities as bundles of local concentrations of activity surrounded by more scattered activity [S2].

More specifically, Figure S1 plots the relative area of the business stratum as observed when varying which census blocks are included in the stratum’s spatial definition. In specific, census blocks are included when harboring, on a block-by-block basis, at least a given threshold number of jobs. When this threshold is higher, fewer census blocks are observed, meaning a smaller total area of the observed stratum.


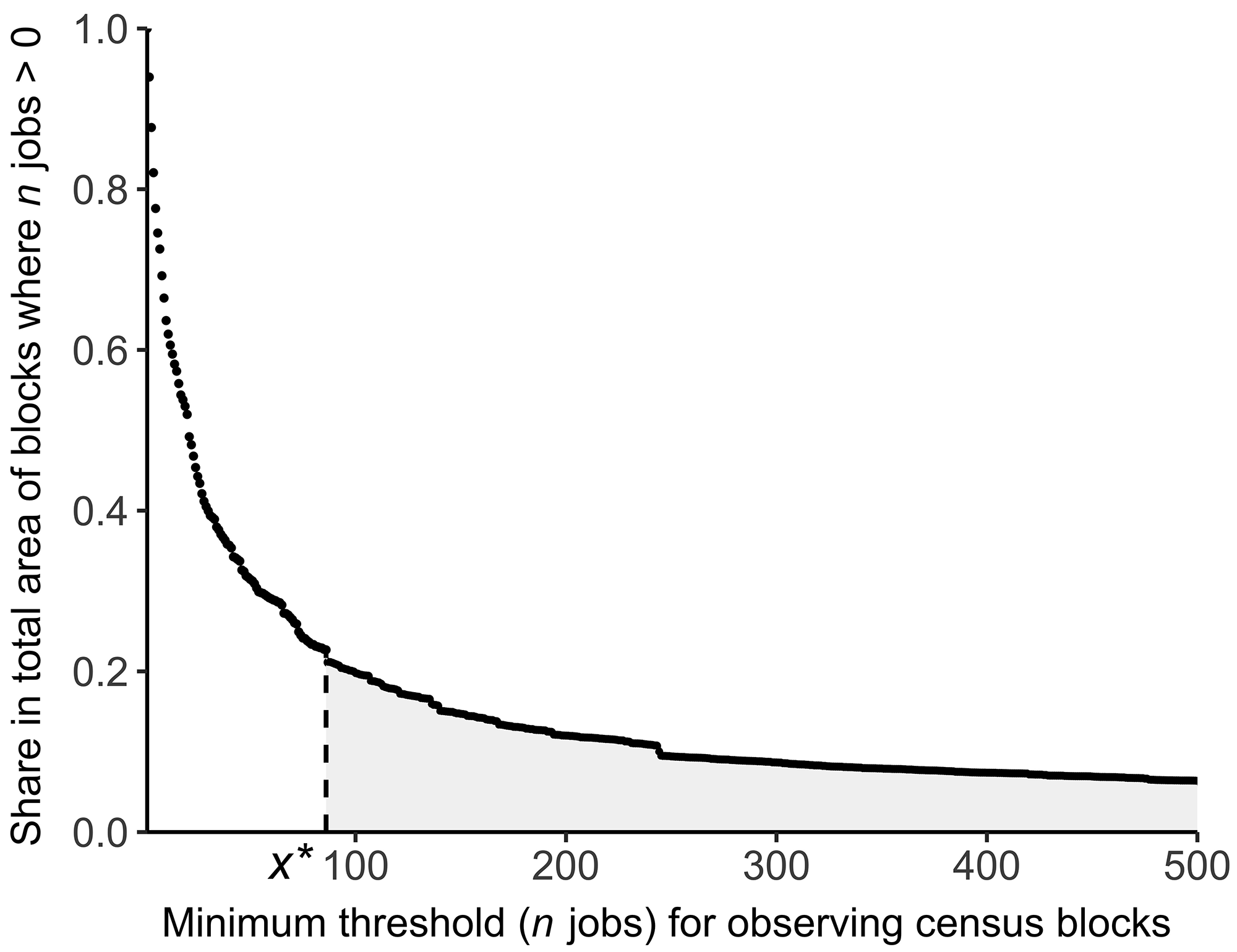


FIGURE S1—Area-efficiency of the business stratum for training pixel sampling.

An area-efficient threshold is obtained from curvature maximization, which is observed along the figure’s *x*-axis at *x**. Left of this mark, blocks are relatively large in area but include relatively few jobs, whereas census blocks right of *x** include more jobs and relatively less ‘excess’ area of land that is not business-related. As such, the final stratum spatially encompasses all of the census blocks that are associated with any of the possible more restrictive strata, which are indicated by Figure 1’s shaded area.

*II. Sample-size evaluation*

To evaluate the training sample’s size based on model performance, for each size-decile of the full-sample 100 stratified random sub-samples are drawn with replacement. On each of these sub-samples, a model is estimated using cross-validation to then predict the class to which each of the sub-sampled pixels belongs. Based on this classification, for each model the so-called F1-score, or harmonic mean, for the user’s accuracy and producer’s accuracy of business land is obtained. The mean values and standard deviations of these F1-scores, by sample-size decile, are visualized in Figure S2.


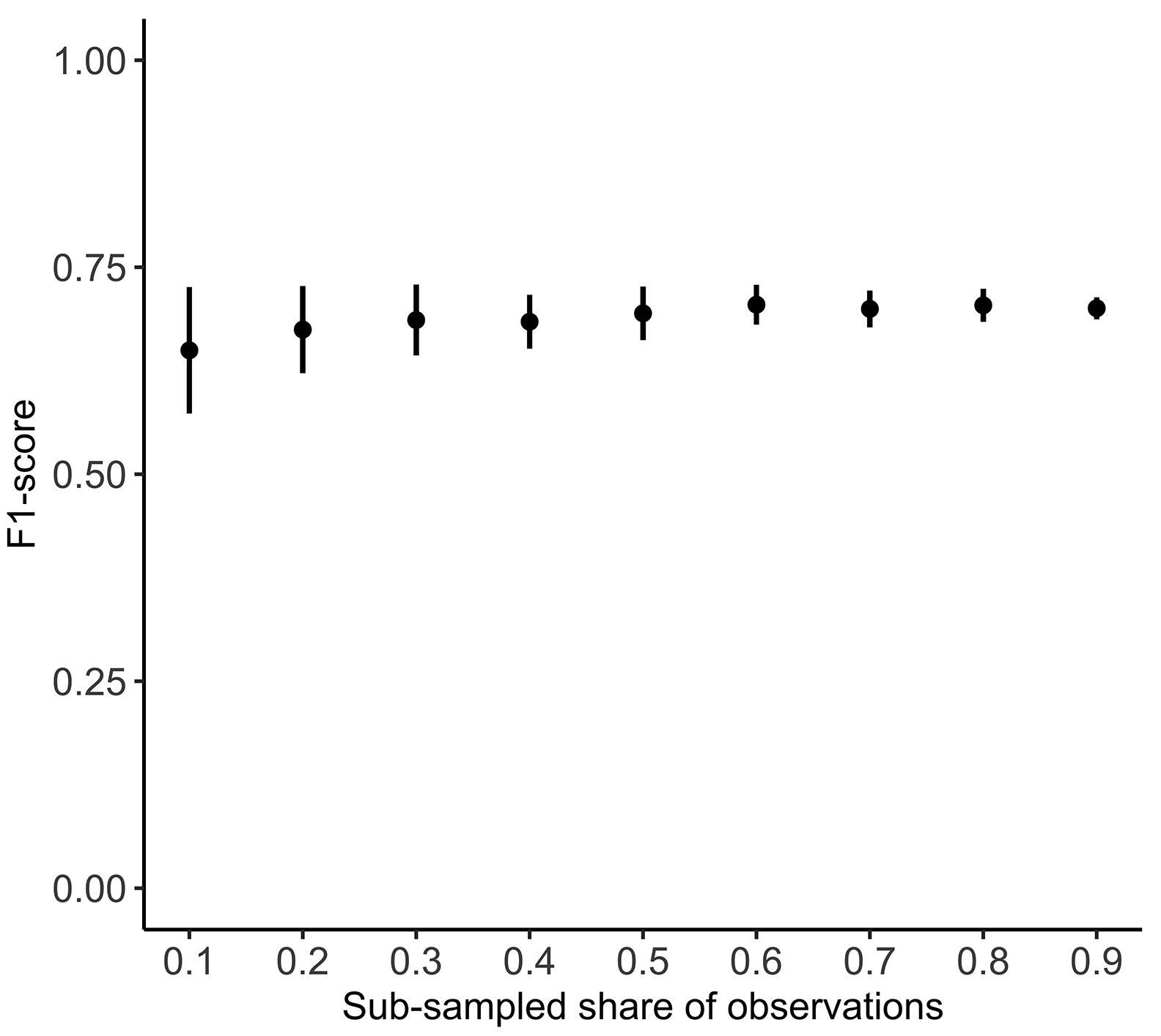


FIGURE S2—Model performance by sub-sample size.

As Figure S2 shows, classification performance stabilizes once the majority of the full sample is observed, with performance for sample sizes close to the full sample’s size appearing to become mostly invariant.

*Supplementary references*

1. Satopää, Ville, Jeannie Albrecht, David Irwin, and Barath Raghavan (2011). “*Finding a ‘Kneedle’ in A Haystack: Detecting Knee Points in System Behavior*,” International Conference on Distributed Computing Systems Workshops, IEEE, 166–171.
2. Ellison, Glenn, and Edward L. Glaeser (1997). “Geographic Concentration in US Manufacturing Industries: A Dartboard Approach,” *Journal of Political Economy*, 105 (5), 889–927.
